# Shaping innovation and coordination of healthcare delivery across boundaries and borders: A comparative case study

**DOI:** 10.1108/JHOM-10-2018-0315

**Published:** 2019-11-07

**Authors:** Rosemary J. Hollick, Alison J. Black, David M. Reid, Lorna McKee

**Affiliations:** 1 Aberdeen Centre for Arthritis and Musculoskeletal Health, University of Aberdeen, Aberdeen, UK; 2 Department of Rheumatology, Aberdeen Royal Infirmary, Aberdeen, UK; 3 Institute of Applied Health Sciences, University of Aberdeen, Aberdeen, UK; 4 Health Services Research Unit, University of Aberdeen, Aberdeen, UK

**Keywords:** Innovation, Complexity, Patient care, Mobile bone density scanning services, Osteoporosis, Healthcare context, Comparative case studies

## Abstract

**Purpose:**

Using a complexity-informed approach, we aim to understand why introduction of a mobile service delivery model for osteoporosis across diverse organisational and country contexts in the UK National Health Service (NHS) met with variable success.

**Design/methodology/approach:**

Six comparative case studies; three prospectively in Scotland using an action research-informed approach; and three retrospectively in England with variable degrees of success. The Non-adoption, Abandonment, Scale-up, Spread and Sustainability framework explored interactions between multi-level contextual factors and their influence on efforts to introduce and sustain services.

**Findings:**

Cross-boundary service development was a continuous process of adaptation and evolution in rapidly shifting healthcare context. Whilst the outer healthcare policy context differed significantly across cases, inner contextual features predominated in shaping the success or otherwise of service innovations. Technical and logistical issues, organisational resources, patient and staff actions combined in unpredictable ways to shape the lifecycle of service change. Patient and staff thoughts about place and access to services actively shaped service development. The use of tacit “soft intelligence” and a sense of “chronic unease” emerged as important in successfully navigating around awkward people and places.

**Practical implications:**

“Chronic unease” and “soft intelligence” can be used to help individuals and organisations “tame” complexity, identify hidden threats and opportunities to achieving change in a particular context, and anticipate how these may change over time. Understanding how patients think and feel about where, when and how care is delivered provides unique insights into previously unseen aspects of context, and can usefully inform development and sustainability of patient-centred healthcare services.

**Originality/value:**

This study has uniquely traced the fortunes of a single service innovation across diverse organisational and country contexts. Novel application of the NASSS framework enabled comparative analysis across real-time service change and historical failures. This study also adds to theories of context and complexity by surfacing the neglected role of patients in shaping healthcare context.

## Introduction

Healthcare systems are increasingly complex, reflecting fragmentation of services across professionals, services and health sectors (Nugus *et al.*, 2010). Effective healthcare delivery requires coordination across multiple boundaries; physical, policy and regulatory, organisational and professional against a background of fast-paced political, organisational and technological change. This paper adopts a complexity-informed approach to understand why introduction of a new mobile service delivery model for osteoporosis across diverse organisational and country contexts in the UK NHS met with variable success.

This paper argues that even apparently “simple” service developments are mired in complexity and illustrates the value of studying comparative healthcare contexts. We reveal how complex interactions, and individual experiences of delivering and receiving care, intersect differently to generate different outcomes in different contexts. Empirical prospective and retrospective, longitudinal case studies reveal practical learning for healthcare professionals and managers to avoid an overly simplistic and linear approach to service improvement. To our knowledge, this is the first empirical research exploring the interplay of system wide contextual factors on the implementation and outcome of osteoporosis services.

The importance of context in healthcare has been recognised for some time, stemming from Pettigrew *et al.*’s (1992) work in the 1980s on inner and outer contexts, and receptive and non-receptive contexts for change. Moving away from a one-dimensional “external” list of factors, multiple levels of context are recognised (Fulop *et al.*, 2005). There is increasing emphasis on the interdependency between human action, organisations, processes and wider system context (Greenhalgh *et al.*, 2004; Mannion *et al.*, 2009; Fulop and Robert, 2015).

However, many studies reveal the continued failure of change initiatives (Dixon-Woods *et al.*, 2012). Simple analyses often fail to capture the complex contexts in which they sit (Hawe *et al.*, 2009) and there is a lack of shared practical learning to support “on the ground” decision making (Marshall *et al.*, 2013). Evaluating too early can mean improvement efforts are unfairly judged as ineffective (Parry *et al.*, 2013) and successful interventions in one healthcare setting can be difficult to replicate elsewhere (Dixon-Woods *et al.*, 2013).

Understanding how interventions work, evolve and adapt over their lifecycle is important to optimise effectiveness in different healthcare contexts (Fulop and Robert, 2015; Marshall *et al.*, 2017). Recent studies are attempting to map this complexity. These explore how multi-level contextual factors interact to shape outcomes of healthcare improvement initiatives (Fulop and Robert, 2015) and challenge the traditional linear, mechanistic models of implementation (Braithwaite *et al.*, 2018).

A number of theoretical frameworks have been developed to further understand and operationalise the study of context: diffusion of innovations (Greenhalgh *et al.*, 2004; Robert *et al.*, 2009), complex adaptive systems (Keshavarz *et al.*, 2010), complexity theory and Normalisation Process Theory (May and Finch, 2009). Most recently, Greenhalgh *et al.* (2017) developed the Non-adoption, Abandonment, Scale-up, Spread and Sustainability (NASSS) framework. Based on empirical case studies and hermeneutic systematic review, this multi-level framework was developed to encourage complex thinking. It incorporates and extends a number of theoretical frameworks to help predict and evaluate success and failure of technology supported health and social care programmes. The NASSS framework explores complexity across seven domains: the condition (clinical and socio-cultural aspects of the condition/illness), the technology (material and technical features), value proposition (if and for whom it represents value), adopter system (staff, patients and carers), organisation (capacity and readiness for change, work of implementation), and wider system (political, regulatory, socio-cultural and professional). The seventh domain explores interactions across domains to embed and adapt interventions over time.

Within each domain, aspects may be considered simple; (following a recipe – predictable and straightforward); complicated (sending a rocket to the moon – still predictable but with multiple interacting components); or complex (raising a child – may encompass both complicated and simple subsidiary problems, but not reducible to either) (Glouberman and Zimmerman, 2002). Complex problems require an understanding of the unique local conditions and are characterised by interdependency, non-linearity and adaptability. By considering complexity within each domain and the interactions between them, a rich narrative of events unfolding in a real-world setting can be generated (Greenhalgh *et al.*, 2018).

However, significant research gaps remain. There is need for more flexible, nuanced and methodologically pluralistic approaches to guide implementation and evaluation of healthcare interventions in a complex, fast-paced healthcare environment (Greenhalgh *et al.*, 2018). Participatory action research (Waterman *et al.*, 2007) and collaborative “researcher in residence” methods (Marshall *et al.*, 2014; Vindrola-Padros *et al.*, 2017) offer the potential for new insights. Similarly, there is increasing recognition of the role of “soft intelligence” to increase the success of improvement efforts (Martin *et al.*, 2015). This can been defined as the processes and behaviours associated with seeking and interpreting soft data to produce useful knowledge to identify problems and find creative solutions (Martin *et al.*, 2015). Yet, all these approaches have been underutilised within healthcare settings. Within the UK, there is a growing divergence of healthcare structure, policy and care provision between Scotland and England. This has created a “field laboratory” ideally suited for comparative analysis and cross-border learning (Steel, 2017). Despite this, very few empirical studies have traced the parallel development of a particular service innovation in both countries (Dayan and Edwards, 2017).

Osteoporosis services are a good exemplar of complex, cross-boundary care. Osteoporosis causes fragile bones which are more prone to fracture. Systems of care for osteoporosis consist of individually “simple” components: identification and assessment of individuals with risk factors for osteoporosis using fracture risk assessment tools and bone density (DXA) scanning to inform treatment decisions. However, delivery of care is complex, requiring coordinated action from multiple health and social care professionals across both community (primary care) and hospital (secondary care) settings (Harrington, 2011).

Mobile bone density scanning (DXA) services were proposed as a “simple” solution to geographical inequalities in access to care for those living in rural communities (Hernlund *et al.*, 2013). Comprising of a whole body DXA scanner housed in a specially adapted van, they aim to deliver services closer to home. Services have been introduced in the USA (Newman *et al.*, 2004), Australia (Tucker *et al.*, 1997) and England in 2008. To date, evaluation has largely focussed on technical comparability of mobile and static DXA scanners (Ulrich *et al.*, 1997) and clinical outcomes (Tucker *et al.*, 1997). Yet, only one of four services introduced in England were successfully embedded into routine clinical practice.

In Scotland, mobile DXA services presented an attractive solution to geographical inequalities in access to services. Creation of mobile services within Scotland provided the opportunity to prospectively study introduction of mobile DXA services. At the same time we explored why similar services had variable success elsewhere and what could usefully be learnt to inform prospective service development.

This paper is based on six comparative case studies, combining both prospective and retrospective longitudinal analysis of the introduction of mobile DXA services across six sites in the UK NHS (Scotland and England). In a novel approach, we have applied the NASSS framework as an analytical tool to compare real-time and retrospective case studies, and generate a rich narrative of events shaping innovation and coordination of healthcare services from multiple perspectives. This paper has two inter-related aims: first, to explore the complexity within each NASSS domain, interactions between domains across the lifecycle of mobile DXA services and how this relates to service implementation outcomes. Second, it aims to reveal practical learning to support “on the ground” decision makers by exploring individual and organisational characteristics that helped or hindered service implementation.

## Methods

### Case selection

Comparative case study selection aimed to facilitate an understanding of how interactions between NASSS framework domains influenced efforts to introduce a new service delivery model; from “conception”, early implementation through to assimilation into routine clinical practice. Using a theoretical replication approach (Yin, 2014), case sampling reflected diverse geographical and country contexts, as well as representing varying stages of the service “lifecycle”.

Mobile DXA services spanning three health boards in Scotland (Island A, Island B and Mainland) were proposed to address unmet clinical need. This presented the opportunity to study service set-up from conception. The mobile DXA service was developed and run by the Scotland Mainland health board and piloted across two remote islands (Islands A and B) and one rural mainland site (prospective case studies).

In England, three out of a total of four similar mobile DXA services were purposively selected for retrospective analysis, based on differing service implementation outcomes; unable to get established (England North), decommissioned after three years (England East) and successfully assimilated into routine practice (England South-West). The key features of each comparative case study (service) are summarised in Table I.

### Data collection

The “revised Conceptual Model of technological adoption and assimilation in healthcare organisations” by Robert *et al.* (2009), subsequently referred to as the revised Conceptual Model, was initially used as a framework to inform data collection. This empirically grounded framework considers the adoption, implementation and assimilation process of technological innovations within healthcare settings. This included the policy and wider healthcare context, organisational context and readiness for change, characteristics of service adopters (patients, healthcare professionals and managers) and features of the technological innovation (in this case the mobile DXA scanning service). Across all cases, we gathered data from site visits, non-participant observations, formal and informal interviews, meetings, telephone conversations and e-mails. The study lead (RH) was also a clinician who led service implementation in the Scotland cases. In the Scotland cases, detailed auto-ethnography (Ellis *et al.*, 2010) by service implementers over a three-year period gathered self-reflections, anecdotal and personal experiences and observations of the service improvement journey. Observations and feedback from patient, public and staff meetings, general practitioner (GP) and patient surveys conducted pre- and post-introduction of mobile DXA services were collected and analysed. This paper is based only on qualitative data collection, including free-text comments from surveys. All formal qualitative interviews were conducted by RH, audio-recorded and fully transcribed, lasting between 30 min and 3 h. Table II summarises the complete data set.

### Data analysis

Three stages of data analysis systematically explored the interaction between multi-level contextual factors across the lifecycle of each service from conception, initial service set-up, implementation and sustainability:

- Stage 1: thematic analysis of the qualitative data was undertaken using the Framework method (Ritchie and Spencer, 1994) and emerging themes identified.
- Stage 2: a systematic deductive approach applied the seven NASSS domains to the emergent themes.

We developed individual case study narratives iteratively with each stage of data analysis. Initially containing rich data on the local healthcare context, gathered from documentary review and site visits, these were expanded to define the problem in each case and a chronology of events. Case narratives supported longitudinal analysis, helping to identify linkages between factors and over-arching analytical themes (Langley, 1999):

- Stage 3: comparative case study analysis compared and contrasted over-arching themes between and within prospective and retrospective cases to explore interactions between multi-level contextual factors (Yin, 2014).

In the prospective case studies, emerging findings were presented and discussed with patients and a broad range of health professionals at regional education and update meetings throughout the study period (2014–2017). Detailed field notes were collected and feedback incorporated into ongoing data analysis. Ethics committee approval was obtained from London and Harrow NHS Research Ethics Committee (15/LO/1946).

### Reflexivity

A reflexive diary was kept by RH throughout the study as a validity enhancing strategy to address some of the methodological challenges posed by the action research-informed approach. Diaries were also kept by radiographers in the prospective cases. Notes from clinical team meetings and engagement with a range of community-based staff and public attending service update meetings gathered evidence from a broad range of perspectives. Findings represent the output of discussions between researchers, staff and public over a period of three years. Sampling and interviewing sought out disconfirming perspectives by selecting patient and staff with a broad range of backgrounds, geographies, experiences of care and roles in service development. Formal interviews were summarised, feedback requested from participants and incorporated into the analysis.

## Findings

Findings are presented in two sections. The first section examines features within the first six NASSS domains and makes an assessment of complexity, illustrated by examples from across case studies. The second section examines interactions between and within domains across successful and unsuccessful services. Factors influencing individual and organisational ability to embed and adapt mobile services over time are explored (domain seven).

### Section 1 complexity within NASSS domains

#### Domain 1 – nature of the condition

Individuals with osteoporosis present to a variety of healthcare providers across primary and secondary care. Osteoporosis was often viewed by health professionals as “someone else’s problem”:

[…]nobody wanted to take it on at all[…]one of the directors of public health told me that [they] didn’t see why we should bother doing osteoporosis at all[…] [GPs] should just put everyone on bisphosphates [treatment][…]and the orthopods [orthopaedic surgeons] were fairly disengaged with the whole thing”.

(Senior hospital clinician, England North)

This contested ownership made it difficult to secure support for cross-boundary service development. The focus on fracture prevention was also a difficult concept for some patients. Whilst many viewed osteoporosis as important, they did not consider it “life threatening”:

It’s not life-threatening, is it? So it doesn’t really matter.

(Patient, Scotland Mainland, 03)

For patients at risk of fracture, some struggled to see the importance of assessment or treatment of a “silent” condition:

[…]people don’t necessarily think that they’ve got a problem until they’ve just had a fracture, so they don’t necessarily see the need[…] they’re cynical about doing things.

(Project manager, England North)

For those experiencing a fracture, loss of mobility and confidence made physically accessing services difficult:

The older you get travelling becomes harder. If it’s icy and cold you don’t want to go out because of fear of falling, especially something like this.

(Patient, Scotland Mainland, 01)

#### Domain 2 – technology

Healthcare professionals initially perceived installation and delivery of DXA services within a specially adapted van as relatively simple, with scanning on the mobile unit technically similar to a static unit. However, when the service was trialled in England, the importance of temperature control of the internal van became immediately apparent. Unless maintained at a constant temperature it was unable to operate. This necessitated provision of an electrical socket at each site:

One particular GP practice wouldn’t let us leave it plugged in overnight[…]we had 2 weeks of ice and snow[…]so we had to cancel patients[…] but that then informed what our policy should be on electricity[…]any location that we go to has to have 24/7 electricity.

(Radiographer, England South, 01)

The logistics of moving the mobile unit across country (and sea) and ensuring a constant electrical supply proved complicated in all cases. New relationships and processes had to be established between transport departments and clinical services:

We have huge problems with moving the mobile unit off site[…]drivers who don’t really know how to drive one of these vehicles[…]problems in the set-up[…]problems in that when the lorry is due for servicing it is not picked up or brought back when we expect it to be done.

(Radiographer, Scotland Mainland, 04)

Poor information technology (IT) intra-operability added to the work of implementation; the mobile DXA scanner was not connected to the main hospital systems so new processes to securely transfer data had to be created. IT incompatibility between patient management systems in particular was a barrier to cross boundary working between public and private healthcare providers in England:

I’m probably running about six different packages for different types of clinic or different specialities[…]they don’t link.

(Community hospital administrator, England South, 03)

All mobile DXA services piloted in the UK were externally charitably funded. Whilst they had been “adopted” by local NHS organisations, mobile units were not viewed as part of the NHS. This led to conflicts regarding responsibility for maintenance and repair of the van and scanner. In one case, this led to the scanner being “off road” for eight months whilst disagreement as to who should pay for repairs was resolved:

[…]with each little hiccup, it took weeks or months to get these things sorted out, because obviously the Trust didn’t want to pay for the repairs because at that point it wasn’t a Trust vehicle.

(Senior hospital clinician, England North)

#### Domain 3 – value proposition

Across all cases, there was an initial assumption that the mobile service was valued equally by all key stakeholders, representing an obvious and simple solution for everyone. However, for the DXA manufacturer, mobile services were a “niche” market, representing little financial gain. In Scotland, agreement to provide onsite technical support whilst scanning on the islands was provided “as a favour”. Temporary withdrawal of technical support during a period of financial constraint created significant anxiety for staff.

Despite fractures representing a significant financial burden to health and social care, and national policy directed towards more integrated, community-based care (The Scottish Government, 2013), osteoporosis competed with a number of other healthcare priorities. The value local organisations attached to developing osteoporosis services varied depending on how they matched local strategic priorities. The prospect of addressing unmet clinical need raised concern about increased prescribing costs and increased demand. For NHS managers, prevention of future fractures was hard to measure and longer-term projected cost savings were difficult to reconcile with pressures for immediate cost savings.

The way service providers and users thought about places and access to services shaped both the value assigned to the service and the effort of implementing change. Addressing geographical inequalities in access to services for those living in rural areas was a primary driver for development of mobile DXA services across all cases. It assumed a shared understanding of the definition and meaning of rurality, defined by distance from urban conurbations, and therefore a shared value of mobile services. Yet, this proved multifaceted and complex.

For many patients in Scotland and England South cases, the mobile service represented the only realistic means of accessing DXA services. The “right kind of rurality” provided a strong rationale and operational mandate for service development. For many staff, it represented an exciting opportunity to develop services in a way that directly benefited their patients and addressed their core beliefs:

It suits our geography and our region very well to have a mobile service […] it was very exciting […] it was the right thing to do and I felt privileged that I was in a position […] to design a service that’s going to help our rural people.

(Senior radiographer, England Southwest, 01)

Patients, that is what we do […] and this mobile is just right, it treats the patients that are unable to come.

(Radiographer, Scotland Mainland, 02)

Similarly, GPs viewed the mobile service as a means of enhancing a wider community ethos of care created by “like-minded GPs and like-minded patients”:

Our population are very keen on expanding services here […] we are equally if not keener on getting more services here so that we can keep people here […] when one thing like the mobile DXA scanning comes in, that shows people that […] the health service is investing time in this population […] Being able to have a collective, almost kind of a community ethos of where you, where the community wants to be.

(GP, Scotland Mainland, 18)

This was counterbalanced by the significant effort involved in delivering care remotely which for radiographers and technicians represented a significant change in working practices (see Domain 5).

Rural patients framed preferences and experience of local healthcare services in terms of social aspects of place. This included quality and time for personal interaction with staff, fear and uncertainty regarding journeys and interactions with friends and family. Whilst staff often viewed remote working as a lonely, isolated experience, many patients described staff as “less distracted” by administrative tasks on the mobile unit and valued more personal interactions, for example, being escorted out to the mobile unit in the car park by a member of staff:

[…] the whole ambience of the rural hospital and rural facilities is a lot less stressful […] you get quicker and better attention in a smaller place […] in [the City] it was very business-like and I felt that I was in a queue.

(Patient, Scotland Mainland, 03)

For many patients, local service delivery meant patients did not have to rely on others to access care, yet for others “a trip into town” provided a welcome opportunity to shop and visit family and friends:

If an appointment comes through that’s at [the City] my, my tummy turns over and I think, “Oh, no, how am I going to get there? Who will take me?” and spending money phoning people asking if they can take me, “Oh, I can’t, I’m working”, and […] then I feel guilty and then I have to, I feel I have to buy something for the person that took me as a thank you […] and it’s money I can’t afford.

(Patient, Scotland Mainland, 01)

However, perceptions of rurality varied across cases. In England East, despite rurality being the main drive for development of the mobile DXA service frontline staff reported that:

[…]there are lots of towns around here so there’s nothing really rural, especially when they’re in one town and have to come here […] they’re not rural.

(Technician, England East, 01)

Patients did not always make decisions when and where to access services simply based on distance. Mobile sites that were physically closer to patients were not necessarily easier for them to access by public transport.

#### Domain 4 – adopter system (staff and patients)

Introduction of mobile DXA services impacted on staff in complex and unpredictable ways; however, the actual implementation process appeared to have been given little attention:

It sort of landed, you know? Then we was like, “Oh, where do we take it from here?”

(Technician, England East, 04)

Mobile DXA service development represented a significant change in scope of practice for staff. Across cases, no existing infrastructure and processes were in place to service and maintain the mobile units. Whilst some transport departments agreed to “adopt” the units, they were often viewed as external to the NHS which brought new pressures; healthcare professionals found themselves grappling with servicing and maintenance schedules for lorries, tachograph cards and regulations around driver hours. Transport staff, used to delivering goods between hospital sites, struggled to understand the clinical aspects of service delivery:

A lot of them see it, “Oh, it’s just like moving a truck from A to B” because they’re not aware of the, I would say the bigger picture really.

(Transport manager, Scotland Mainland, 17)

The technical and logistical efforts required to move the unit across the country brought to the fore inter-departmental tensions. This was managed by forging personal relationships and “pulling in favours”, which was hampered by frequent reorganisation at operational management level and changes in personnel.

For radiographers and technicians, the mobile services represented a significant change in working practices and conditions. Not all staff relished the prospect of scanning in a different location. Adjustments to working practices such as travelling to work in a different location or living remotely for short periods of time when delivering services to the islands impacted on travel arrangements to and from work, caring responsibilities and other social activities. Working remotely meant it was impossible to do other administrative tasks when, for example, a patient failed to attend their appointment. All this influenced staff actions in unpredictable ways. Whilst some staff loved the freedom it gave them, others hated it:

It was cold, it was wet and damp. It was a pain in the backside.

(Technician, England East, 06)

How patients felt about places also shaped efforts to implement services. The decision about where to locate the rural mainland pilot in Scotland was between two small towns situated 10 min apart. In the chosen site, the population described themselves as “country people” keen to develop and use local services. In contrast, the neighbouring town was described by staff and patients as having an “urban” mindset and less likely to use local services:

Our population very much view themselves as country people, but not ten minutes away […] [they] very much see themselves as urban commuting belt rather than out in the country […] I’m not sure where the magic cut off is.

(GP, Scotland Mainland, 18)

Introduction of the mobile service to Island B stalled due to concerns about increased prescribing costs. Angered by the fact that Island A had access to the service, whereas Island B did not, patients on Island B independently mobilised a “groundswell” of support within the community. The resulting political pressure was key to “gaining traction” to establishing the service on Island B. In England East, patient preferences regarding use of the mobile vs static service resulted in significant scheduling problems as the service attempted to make best use of staff time and fill scanning lists at peripheral sites. A typical conversation was described:

“Well, my friend went on the mobile and that’s better for me, why can’t I? […] I don’t want to come to [City], I’d rather go to [rural Town]” […] or “I don’t want to go out there, can I come to [the City] because I want to come and do my shopping?” […] or “I don’t drive so I can’t get there [mobile site]” […] it might only be two miles from where they lived but there was no public transport going across.

(Technician, England East, 01)

#### Domain 5 – organisations

Support for mobile services varied across their lifecycle as local priorities shifted in response to wider political pressures, services were restructured, personnel changed and resources were squeezed. It was difficult in some cases to “sell” future financial savings in fracture reduction when the immediate pressures were on meeting annual budget targets (see Domain 3):

Unless they’re going to save money immediately the commissioners won’t even consider it.

(Senior radiographer, England South, 01)

Health boards are held to account over their annual finances […] you’re fire-fighting all the time but you’re not actually investing in infrastructure change […] there is a rigidity of thinking, and I think that’s partly driven by the targets we’re gonna be judged on.

(Senior Clinical Manager, Scotland Islands, 05)

Across cases, organisational infrastructure and processes had to be created to support mobile DXA services. External charitable funding created an additional layer of logistical complexity due to lack of integration with NHS administrative processes. Staff had to “figure it out” as they went along, such as organising ferry travel for the van and flights:

You’re going in with blinkers until you physically try and arrange a visit[…]and do a visit before you realise just what is all involved for the set-up, getting there, dealing with it, planning it, everything.

(Administrator, Scotland Mainland, 09)

Across cases, there was significant variability in how osteoporosis services were configured within each organisation which appeared to directly impact on their stability and capacity for service innovation. The quality of operational support for services also varied considerably across cases. Building build rapport and new relationships between clinicians and managers took time, and repeated reorganisations at operational level was reported to have a negative impact:

To develop and build a relationship and understand one another it takes a bit of time for that to happen and to bed in and for the whole team especially to get to know them...so yeah, frequent reorgs have a huge impact.

(Hospital manager, Scotland Mainland, 01)

Staff reported that smaller services were also “deprioritised” and competing for time and resources, often finding themselves in sharing a manager who was also responsible for a much larger service:

It’s bolted on to [medical specialty] and it’s not got proper push behind it[…]for one period we were being co-managed by the person who was also managing A&E[…]they are basically just swamped and if they’ve got 5% of their time available for you that is it.

(Senior hospital clinician, England North, 02)

In all cases, services were reliant on one or two key champions which left services vulnerable. Personal relationships between individuals appeared to transcend established procedures and policies to enable things to get done, or not:

I have got a very almost unique position to be able to influence change because of my longevity within the organisation[…]it’s not what you know, it’s who you know that counts.

[Senior clinical manager, Scotland Islands, 04]

Reductions in national waiting time targets for DXA made it difficult to gather enough referrals from sparsely populated areas within the given timeframe to justify sending a mobile service. Whereas some organisations were willing to apply these targets flexibly to mobile services, others were not. In England Southwest and Scotland cases, as long as patients had been offered an appointment within the waiting time target, if they chose to wait longer for the next visit of the mobile service that was a deemed a “legitimate patient choice”. However, in England East trust the national waiting time target was rigidly adhered to with no exceptions.

#### Domain 6 – the wider system

Changes in national policies had varied effects on service implementation. For example, during early service development in Scotland, Scottish Government transferred central funding for subsidised travel from rural areas to access healthcare to local health boards. This was intended to incentivise “repatriation” of services to the islands by enabling health boards to keep any cost savings made.

In NHS England, competition has been used to drive up quality and standards, with a “purchase/provider split” creating formal contracts between care commissioning groups and providers of healthcare services (Cylus *et al.*, 2015). The complex milieu of NHS and private healthcare providers presented significant challenges to development of cross-boundary services such as mobile DXA, in particular IT incompatibility between different provider patient management systems. Contrastingly a more integrated, partnership approach to healthcare delivery in Scotland (The Scottish Government, 2011) was reported to facilitate cross-boundary service development:

We still have a pretty joined-up NHS in Scotland operating without accountants trying to determine whether each service is introduced makes it an ideal place to test it and easier to introduce it.

(Senior Hospital Clinician, Scotland Mainland, 11)

Historical relationships between healthcare providers also influenced patterns of cross-boundary working over and above organisational and commissioning boundaries in unpredictable ways. Understanding and leveraging these links using “soft intelligence” proved key in securing access to key peripheral sites for the mobile DXA services.

Features of the wider healthcare context also generated insecurities around longer-term sustainability of mobile DXA services which had to be actively negotiated. In England, the commissioning process reportedly generated insecurity and acted as a “disincentive” for future service innovation given the significant time and resources invested in setting up new services:

If they [independent provider] say, “Well, we’ll do it for £30 a head” instead of what the tariff is that we get paid, then the commissioners could commission them to provide all the DXA scans and we would be out of business.

(Community Hospital Administrator, England Southwest, 03)

In the Scotland cases, island health boards were reliant on larger mainland health boards for provision of key services. These “geographies of power” also created a palpable sense of insecurity, dependence and lack of control over service development within the island health boards:

You’re like this big fat banker almost, and the little ones are a bit like, “Please, sir, can I have some more”.

(Hospital Manager, Scotland Mainland, 01)

We’re a very small part of [Scotland Mainland] business […] they don’t have to take any notice of us because we are small beer.

(Senior Clinical Manager, Scotland Islands, 01)

### Section 2 characteristics of successful vs unsuccessful services

In this section, we first consider how key NASSS domains interacted to influence service outcomes across successful and unsuccessful cases, in particular the unpredictable and emergent interactions between staff and patients. We then consider the factors influencing the ability of individuals and organisations to embed and adapt mobile services over time.

Individual case study narratives indicated how complex and unpredictable interactions between NASSS domains at different time points shaped services outcomes. Cross-case analysis, see Table III, revealed that successful and successful cases differed in key domains, with the greatest variability across the technology, value proposition, adopter system and healthcare organisation domains.

#### Successful cases

Service innovation took place within well-established osteoporosis services. The stable configuration and set-up of local osteoporosis services appeared critical in developing and supporting key service champions with strong professional networks and organisational memory. These champions were well placed to sense opportunities in the wider context such as charitable funding to support service set-up. Similarly, in the Scotland cases, changes to a national travel reimbursement scheme enabling island health board to keep costs savings realised by “repatriation” of services from the mainland were timely in gaining early support for creation of a mobile service. During the early adoption process, champions played a key role in navigating the complex mix of private and public healthcare providers, relationships and “geographies of power” between organisations, gathering support and creating a shared vision across diverse stakeholders. Staff experienced in bone densitometry were able to identify the necessary technical and logistical support needed for the mobile unit.

However, high levels of operational management turnover at the time of early service set-up in the Scotland cases hampered timely decision making to enable installation of electrical sockets and unit transportation. Yet, experienced and well-networked staff used personal contacts to bypass this “layer of inertia”. In contrast, service roll out initially faltered after early pilot work in England Southwest due to poor project leadership. Alert to the problem, operational managers proactively intervened with additional support before appointing a new service lead. Within both organisations, flexible application of national waiting time targets to mobile services was critical to support pragmatic service scheduling.

The perceived degree of rurality provided a strong rationale and operational mandate for a mobile DXA service from both patients and staff, and fitted with core beliefs to deliver better care for patients. Positive patient experiences shaped the value staff attributed to the service, over and above the considerable work of implementation and personal effort to deliver care remotely. This added to a growing sense of positive momentum around services. Patients were also influential in shaping the actions of organisations and the wider political context, as in the case of Island B where patient lobbying of both the local health board and politicians rapidly resulted in a formal agreement to implement the service.

Embedded service evaluation provided real-time feedback to staff on patient experiences and outcomes which was used to inform ongoing service development and provide evidence to secure long-term funding. In England Southwest, the trust was creative in their approach to commissioning the mobile service at the end of the pilot period, incorporating it as part of the overall osteoporosis service rather than a separate service. This rendered it less vulnerable to competition from external healthcare providers.

#### Unsuccessful cases

Key individuals within local osteoporosis services had championed mobile DXA services within regional osteoporosis strategy groups and secured local and national charitable funding for a three-year pilot. However, prior to delivery of the mobile unit, the service in England East “imploded”. Organisational service restructuring moved the well-established service into a different directorate, resulting in the loss of key service champions and an additional clinical workload for remaining staff. In England North, whilst attention was focussed on obtaining commissioning support for the proposed osteoporosis risk assessment programme, the existing osteoporosis service was undeveloped. Whilst sitting in a high achieving organisation, osteoporosis was not a strategic priority. Perceived as a “bolt-on” to the medical specialty in which it sat, the service lacked permanent staff with specialist training in bone scanning.

In both cases, this left service implementation to staff with little capacity for service development. In England North, whilst staff and patients reportedly placed high value on the creation of a mobile DXA service for a large rural population, there was no “dedicated” DXA team with the appropriate knowledge and skills. Consequently, many the technical and logistics aspects of service delivery were neglected or misunderstood, for example, ensuring a constant temperature in the mobile DXA unit by means of a permanent electrical supply. Failure to overcome the technical and logistical issues resulted in a spiralling sense of frustration enveloping the service and the mobile service appeared to have been “written off” as unworkable by management. Despite gaining commissioning support for the service, the implementation process petering out without the service becoming operational.

In England East, the new service lead lacked the well-developed professional networks of their predecessors and was unable to secure access to key rural sites of the small trust catchment area. Vulnerable to competition from private care providers and neighbouring trusts, these sites purchased DXA services elsewhere. Despite experienced bone densitometry staff, organisational failure to support the technology requirements of the mobile service left the unit unplugged and cold overnight. The need for a prolonged warm up period in the morning reduced scanning time on the mobile unit.

Patients were also reportedly ambivalent about the service and unpredictable patient decision making to access the mobile or static services compounded existing service scheduling issues. The increasing work of implementation appeared to deter staff from seek out alternative rural sites. This combined with rigid adherence of the trust to the new six-week national waiting time target for DXA resulted in the newly established mobile service making more frequent visits to “less rural” sites. For many frontline staff, already questioning the value of the mobile service in small trust where nowhere was “that rural”, the service was perceived as increasingly inefficient and “pointless”.

Whilst plans had been made in the pre-implementation phase to “work the system”, identifying and referring patients in “batches” to overcome the problem of waiting time targets, organisational restructuring had left remaining staff with no contacts in primary care to enact this. Constant benchmarking of the mobile service against the established static DXA service in England East appeared to foster a rigid, non-adaptive approach to service implementation. Staff reported a moral obligation to use charitable funds for their intended purpose which hindered adaptation of the mobile service model in response to evolving challenges.

All these factors conspired to create a spiralling sense of negativity around a service which “just became harder and harder to work”. No evaluation was undertaken in England East. The organisation submitted a commissioning bid for the mobile as a separate (and more expensive) service. This was rejected and the service was actively decommissioned by the trust after three years.

#### Managing complex interactions between domains

Flexibility and adaptability at both individual and organisational level were key to managing the complex interactions between domains. Setting up the new service was an evolving process of trial and error.

Some staff embraced this uncertainty and thrived on the challenge whereas others struggled. The extent to which staff had ownership and autonomy to adapt services varied between cases, shaped by resources, professional hierarchies and system constraints:

There is a little bit of serendipity […] you needed a chance when it comes up and that connects with something else I’d like to do and let’s see if we can do it.

(Senior Hospital Clinician, Scotland Mainland, 11)

Some radiographers felt less empowered than doctors to enact change. Charitable funding, whilst enabling creative clinicians to undertake service development which would otherwise have been difficult within NHS constraints, created other constraints:

How can we say “we don’t want this but we want something else?” I don’t think anybody was keen to tell [the local charity] for the first sort of year or so that it wasn’t working.

(Technician, England East, 04)

Ongoing conflicts over responsibility for transportation, repairs and cleaning of the mobile van hampered integration into NHS organisations, and persisted even in successful services.

In successful cases, a flexible “mindset” facilitated iterative evolution of services to meet demands and adapt to external challenges:

[…] what the service looks like now isn’t how I envisaged it to look at all […] I had a plan that we would have a regular schedule […] but the referral patterns didn’t reflect that […] so it did evolve.

(Senior Radiographer, England Southwest, 01)

Plans and processes agreed in the early stages of service development were regularly reviewed, renegotiated and new “workarounds” developed in light of ongoing feedback. Staff reported that they were “constantly looking over their shoulder”. Despite being unable to get the service established in England North, the project manager used networks developed with the mobile service to improve other aspects of the service, including training and education.

Auto-ethnographic data from the Scotland cases also provided rich insights into the importance of generating and sustaining the motivation of key stakeholders throughout the lifecycle of services at different organisational levels, from the van driver to chief executive. This required constant attention. “Softer” management skills were important in maintain staff support and anticipating and attending to what could be perceived as trivial issues, which if neglected could have significant consequences. For example, issues with cleaning of the van interior at one site threatened staff willingness to scan at that location and undermined hard won relationships with community-based partners. Personal relationships and tacit knowledge of awkward people, who to work around, over or under to get things done were essential to effect change.

Comparative case study analysis revealed the importance of evaluation in adapting and embedding service change. The retrospective cases studies uncovered a wealth of useful information that critically informed service development in the Scotland cases, yet no learning was shared between the three retrospective cases at the time of service development. Staff were understandably reluctant to be judged as a “failed” service, especially when charitable money had been used to set-up services.

## Discussion

A complexity-informed approach revealed rich insights into coordination of care across boundaries and borders. Service development was a continuous process of adaption and evolution in a rapidly shifting wider healthcare context. Technical and logistical issues, organisational resources, patient and staff actions combined in unpredictable ways to shape the lifecycle of service change in an iterative process. Whilst these interactions could produce valuable, new capabilities and “gathering momentum” in successfully sustained services, they could also conspire in a spiralling sense of negativity in services that were ultimately decommissioned.

Novel application of the NASSS framework provided a standardised tool to analyse a diverse data set. This revealed insights into the complex interaction of factors influencing a single service innovation across real-time service change and historical failures. This ex-post theorising (Dixon-Woods *et al.*, 2011) generated new insights into individual and organisational behaviour. It provided a rich, yet underexploited, opportunity to explored why similar services had failed elsewhere, reveal common issues of complexity across diverse healthcare contexts and prospectively inform service change in Scotland.

The auto-ethnographic narrative and longitudinal perspective captured the complexity of contexts in action, revealing the “in flight” and often nuanced relationships between contextual factors, soft intelligence and dogged determination needed to work around awkward people and places. The self-reflexive approach also enhanced healthcare professionals learning, facilitating a better understanding of the service development process and how their actions shaped the context in which they worked (Leslie *et al.*, 2014). Longitudinal analysis also revealed the complexity of judging “success”, recognising that “successful” cases today could be “unsuccessful” tomorrow.

However, as authors RH, AB and DMR were also members of the clinical team in Scotland, there was a risk that their perspective could be influenced by clinical status and vested interests. In the prospective cases, evidence was therefore gathered from a broad range of perspectives; diaries were kept by radiographers, and notes taken at clinical team meetings and service update meetings attended by a range of community based staff, patients and public over the course of mobile service development. Findings represent the output of discussions between researchers, staff and public over a period of three years. However, the need for anonymity in writing up the data has meant that some granular details have been omitted. Within the embedded action research-informed role, there was also tension between a commitment to scientific rigour, and competing demands generated by the organisation and other staff members (Vindrola-Padros *et al.*, 2017). Care was taken to preserve professional relationships, whilst maintaining research integrity and rigour.

Combining prospective and retrospective studies presented both challenges and opportunities. Data collection was more limited in the retrospective cases. For example, direct patient and primary care perspectives were unavailable, and healthcare managers were not available in all cases. Some historical service documentation was not available for analysis and there may have been an element of recall bias in participants’ recollection of past events. However, comparative longitudinal analysis of prospective and retrospective case studies provided a unique opportunity to study introduction of similar services with varying fortunes across different healthcare contexts within the UK NHS. The NASSS framework provided a standardised tool to analyse a diverse data set and capture rich insights that would otherwise have been lost in the retrospective case-studies.

The complexity-informed approach represented a useful conceptual framework for change (Braithwaite *et al.*, 2018). Our analysis revealed the importance of continuous adaption and co-evolution of nested systems (Keshavarz *et al.*, 2010), emergent features and generative relationships (Plsek and Wilson, 2001) in shaping service development. In line with other recent studies, it has revealed the influence of power, politics and social influence on stakeholder interactions (Robert *et al.*, 2010) and the interplay between clinical teams and organisational capabilities on service innovation (Turner *et al.*, 2018). Whilst the outer healthcare policy context differed significantly between the devolved healthcare systems in Scotland and England, there were more similarities than differences in the emergent themes across cases. Inner contextual features such as local actors, service set up and stability appeared more powerful than outer contextual factors in shaping the success or otherwise of service innovations.

Opportunity and problem sensing, alongside flexible thinking were key to successful implementation. At service conception, individual ability to sense opportunities in the wider context was important, however, sustained vigilance for emerging problems, however small, at macro, meso and micro levels was essential to enable successful adaptation and evolution of services. Anticipating potential problems enabled staff to “stay several steps ahead”. “Soft” data, such as an intimate working knowledge of individuals and organisations, and “softer” management skills proved useful in sensing problems and opportunities and finding context-sensitive solutions to navigate around awkward people and places. Organisations were key to creating a supportive environment, acting as a “buffer” to the fluid political context and providing operational managerial support to mobilise the professional knowledge and expertise of frontline staff. In particular, flexibility to accommodate the technical requirements of the new service and mitigate ongoing challenges in the wider policy context emerged as a key determinant of success.

These findings align with the concept of chronic unease which is said to characterise high reliability organisations (Reason, 1997; Flin, 2017). Key features of chronic unease include anticipation of failure, concern about future events and constant vigilance for environmental opportunities and pitfalls. These have been shown to facilitate early detection of problems, prevent over-simplification and encourage flexible thinking to creatively solve problems (Flin and Fruhen, 2015). Applying the concept of “chronic unease” and “soft intelligence” in a healthcare setting provides a useful theoretical framework to consider complexity within healthcare. Whilst this approach has often focused on revealing latent problems, we have demonstrated that “chronic unease” and soft intelligence can also be used positively to help individuals and organisations “tame” complexity and unpredictability, both in a sense-making and problem solving capacity.

This paper has added to theories of context and complexity by surfacing the neglected role of patients in shaping the complex healthcare context (Adeosun *et al.*, 2017; Locock *et al.*, 2019). Our analysis has revealed that patients were active participants in shaping context at all stages of service development, and in different ways across cases. Rather than a single homogenous group, the actions of patients could be enabling, disabling, emergent and unpredictable at multiple points in the service lifecycle. Staff and patients actions evolved over time and combined to shape service development.

This study has a number of practical implications. Frontline staff, healthcare managers and policy makers would benefit from greater understanding of complex interactions shaping cross-boundary care delivery. At the outset of service development, it is important to identify hidden threats and opportunities to achieving change in a particular context, and anticipate how these may change over time. Understanding how patients think and feel about where, when and how care is delivered for a particular condition is essential. A stable base service, strong clinical leadership and development of clear, relevant goals across a diverse group of stakeholders it necessary to navigate through a rapidly evolving healthcare context.

Across the lifecycle of service innovation, attention needs to be paid to factors that may conspire to thwart or support service development; whether the service continues to meet the needs of patients, the ongoing influence of patients on staff perceptions of service value, and maintaining the “will and skill” of staff. This can be supported by routinely embedding evaluation within service development. For policy makers, recognising the unintended and sometimes conflicting impact of national healthcare policies on service development is important to support frontline efforts to develop services sensitive to local needs.

Although this study is set within the UK NHS, it has uniquely traced the fortunes of a single service innovation across diverse organisational and country contexts. Despite the differing political landscapes across the devolved healthcare systems, inner contextual factors were critical in shaping innovation and coordination of healthcare delivery. The NASSS framework provides a useful tool to facilitate comparative analysis and cross border learning. Future work using this framework could explore the development and sustainability of other service innovations across wider healthcare contexts. Within this, embedded, longitudinal clinical research using action research-informed approaches has the potential to bring fresh insights to enhance organisational and complexity research and shape policy making and practice. Patients are integral to future healthcare organisational research, providing unique insights into currently unseen aspects of context. Future work is required to explore how individual staff and patient interactions shape the healthcare context, development and sustainability of healthcare interventions over time. The concepts of “chronic unease” and soft intelligence also provide potential new insights into individual and organisational behaviour. Further empirical research is needed to conceptualise and operationalise these within a healthcare setting and develop practical tools to support innovation and coordination of care across increasingly complex healthcare settings.

## Figures and Tables

**Table I:** Comparative case studies

Case	Geographical context	Service outcomes	Timeframe for analysis
Scotland Island A			
Scotland Island B	Remote islands	Successfully running for 4 years	Conception to full delivery (2014 to 2017)
Scotland Rural mainland	Predominately rural with remote regions		
England North	Predominately rural, with remote regions	Unable to get established	2008–2011
England East	Accessible rural region surrounded by larger urban areas	Decommissioned after 3 years	2009–2012
England South-West	Predominately rural with remote regions	Successfully sustained	2008–present

**Table II:** Data sets for each case study

Scotland cases	Observations and informal interviews 3 years experiential and observational data (RH and 3 radiographers) 10 site visits 13 patient support group meetings, GP and patient education sessions 200 informal interviews (patients, healthcare providers, managers, administrative and transport staff, local politicians) Formal interviews 8 senior managers, 13 clinicians in primary and secondary care, 3 radiographers, 2 nurses, 3 transport staff, 8 patients Documentary sources Over 500 e-mails relating to service set-up National and local policy documents, strategy and business cases Free-text comments from survey data 74 general practices across 3 health boards (75% response rate) 202 patients attending osteoporosis service over one month pre-introduction of mobile (99% response rate) 370 patients scanned during 1st year of mobile service (75% response rate)
England cases	Observations and informal interviews 3 site visits (3 days each) 20 informal interviews Formal interviews 2 senior managers, 3 clinicians, 1 specialist nurse, 8 radiographers/technicians, 2 administrative staff Documentary sources National and local policy and strategy documents Business cases, charitable funding documents, minutes strategy group meetings

**Table III:** Key features of successful and unsuccessful cases

Domain	Key features	Successful cases	Unsuccessful cases
Condition	Contested ownership of osteoporosis services	High levels	High levels
Technology	Support for logistical and technical requirements of mobile DXA service	Generally strong	Poor
Value proposition	Prioritisation of osteoporosis services in context of local and national healthcare priorities	High priority	Variable priority
	Perceptions of rurality and shared value of mobile services	Overall consistent	Variable
Adopter system	Impact on staff working practices	Significant	Significant
	Presence and continuity of key service champions	Strong	Variable
	Cross-boundary communications and influence	Strong and consistent	Variable quality and patchy
	Patient perspective on service development	Overall positive	Mixed
Health/Care organisation	Configuration and stability of local osteoporosis services	Stable and well established	Unstable or under-developed
	Quality of local operational management support	Variable	Variable
	Existing infrastructure and processes to support mobile services	Absent	Absent
Wider system	Coherence of national healthcare policy	Poor	Poor
	Complexity of relationships between providers	High complexity	High complexity
Embedding and adapting over time	Individual and organisation adaptability and flexibility	High levels	Poor
	Service evaluation, feedback and learning	High levels	Poor
